# Spatiotemporal local and abscopal cell death and immune responses to histotripsy focused ultrasound tumor ablation

**DOI:** 10.3389/fimmu.2023.1012799

**Published:** 2023-01-23

**Authors:** Ashley L. Pepple, Joey L. Guy, Reliza McGinnis, Amy E. Felsted, Brian Song, Ryan Hubbard, Tejaswi Worlikar, Hannah Garavaglia, Joe Dib, Hannah Chao, Nicoleen Boyle, Michal Olszewski, Zhen Xu, Anutosh Ganguly, Clifford S. Cho

**Affiliations:** ^1^ Department of Surgery, University of Michigan Medical School, Ann Arbor, MI, United States; ^2^ Research Service, Ann Arbor VA Healthcare, Ann Arbor, MI, United States; ^3^ Department of Biomedical Engineering, University of Michigan, Ann Arbor, MI, United States

**Keywords:** ablation, immunity, tumor, histotripsy, ultrasound, necroptosis, ferroptosis

## Abstract

**Introduction:**

Histotripsy is a novel focused ultrasound tumor ablation modality with potent immunostimulatory effects.

**Methods:**

To measure the spatiotemporal kinetics of local andabscopal responses to histotripsy, C57BL/6 mice bearing bilateral flank B16 melanoma or Hepa1-6 hepatocellular carcinoma tumors were treated with unilateral sham or partial histotripsy. Treated and contralateral untreated (abscopal) tumors were analyzed using multicolor immunofluorescence, digital spatial profiling, RNA sequencing (RNASeq), and flow cytometry.

**Results:**

Unilateral histotripsy triggered abscopal tumor growth inhibition. Within the ablation zone, early high mobility group box protein 1 (HMGB1) release and necroptosis were accompanied by immunogenic cell death transcriptional responses in tumor cells and innate immune activation transcriptional responses in infiltrating myeloid and natural killer (NK) cells. Delayed CD8+ T cell intratumoral infiltration was spatiotemporally aligned with cancer cell features of ferroptosis; this effect was enhanced by CTLA-4 blockade and recapitulated *in vitro* when tumor-draining lymph node CD8+ T cells were co-cultured with tumor cells. Inoculation with cell-free tumor fractions generated by histotripsy but not radiation or freeze/thaw conferred partial protection from tumor challenge.

**Discussion:**

We propose that histotripsy may evoke local necroptotic immunogenic cell death, priming systemic adaptive immune responses and abscopal ferroptotic cancer cell death.

## Introduction

There is great interest in developing ablative therapies to trigger systemic anti-tumor immune responses that could augment the efficacy of cancer immunotherapy (1–13). By initiating immunogenic cell death pathways (e.g., necroptosis, pyroptosis) that release tumor-specific antigens within the context of pro-inflammatory danger signals, ablation could initiate the cancer immunity cycle of antigen presentation and adaptive immune stimulation (14). The cytotoxic capacity of CD8+ T cells primed by checkpoint inhibition immunotherapy has recently been shown to be mediated through ferroptosis, a pathway of oxidative programmed cell death to which cancer cells appear to be uniquely susceptible (15–20). Ideally, immunostimulatory ablative therapies capable of triggering ferroptotic anti-tumor CD8+ T cell responses would be particularly attractive for future clinical translation (10). Focused ultrasound (FUS) ablation modalities using high intensity ultrasound pulses to cause destructive tissue cavitation have shown immunostimulatory promise (21–32). We have observed that histotripsy, a non-thermal mode of mechanical FUS (33–36), incites potent local and abscopal anti-tumor immune responses that are stronger than those generated by thermal ablation or radiation (37, 38). We reported that histotripsy induces the early release of immunogenically intact tumor antigens and pro-inflammatory damage-associated molecular patterns (DAMPs) like high mobility group box protein 1 (HMGB1) within the tumor ablation zone. Using flow cytometry, we demonstrated that these changes were followed by influx of innate and adaptive immune cell populations into treated tumors (37). To understand the spatiotemporal evolution of these changes in treated and distant, untreated tumors, we now report microscopy-based observations of the local and abscopal inflammatory and immune cell responses and cancer cell death pathways that follow histotripsy tumor ablation.

## Methods

### Mice, cell lines, and tumor inoculations

Male and female C57BL/6 mice aged 6–8 weeks old were purchased from Taconic (Hudson, New York) and housed and maintained in specific pathogen-free conditions. Experimental groups were assigned prior to all interventions without randomization and investigators were not blinded to experimental groups. Each experiment involved 4–10 mice per experimental group, and experimental group sizes are noted in the Figure Legends. B16F10 and Hepa1-6 cells were purchased from ATCC (Manassas, VA) and used within 10 passages from receipt. Cell lines were maintained using methods previously described (37). Flank tumors were established by subcutaneous injection with 5-10x10^4^ B16F10 or 5x10^6^ Hepa1-6 cells suspended in phosphate buffered saline (PBS) and Matrigel (Gibco, Life Technologies Corporation) at a 1:1 ratio. Flank tumors were monitored and measured with electronic calipers at least every 3 days, and tumor volumes were calculated according to the following formula: volume=long dimension x short dimension x (short dimension/2). Endpoint criteria for euthanasia included maximal tumor diameter >18 mm with concomitant >50% total body surface area tumor ulceration.

### *In vivo* tumor treatment

Tumors were treated with sham or histotripsy ablation or radiation using protocols previously described (37, 38). Briefly, histotripsy ablation was performed using 50 histotripsy pulses at 100 Hz pulse repetition frequency (PRF) delivered at each location to generate an estimated −30 MPa peak negative pressure at the focus. Histotripsy ablation times ranged from 4-15 minutes. Tumor irradiation was performed by placing mice in radiation chambers with lead body shielding leaving only the tumor exposed to 15 Gy unfractionated radiation. Checkpoint inhibition immunotherapy was administered using methods previously described (37), with 200 μg anti-CTLA-4 mAb (Bio X Cell, Lebanon, NH) administered on selected days *via* intraperitoneal injection.

### Tumor dissociation

Tumors were dissociated into single cell suspensions in collagenase/hyaluronidase (STEMCELL Technologies, Vancouver, Canada) for approximately 40 minutes at 37°C using an Octodissociator with gentleMACS C-tubes (Miltenyi, Bergisch Gladbach, Germany). Cells were then passed through a 70 μm filter before adding 1X ammonium chloride buffer (Stemcell Technologies) to remove red blood cells. Cells were resuspended for 2 min in 0.25% Trypsin-EDTA (Gibco, Life Technologies Corporation) and triturated to achieve single cell suspensions, then washed with PBS containing 2% fetal bovine serum (FBS). Cells were suspended in DNAse I (STEMCELL) for 5 minutes and washed with PBS containing 2% FBS. Cells were filtered through a 40 μm filter before flow cytometry.

### Multicolor immunofluorescence

Multicolor immunofluorescence analysis of tumor cell death and tumor-infiltrating cell populations was performed using methods previously described (37). For cell population analysis, samples were washed and incubated with rabbit anti-mouse anti-CD8a antibody (Abcam, Boston, MA) followed by Alexa 555 goat anti-rabbit secondary antibody (Invitrogen, Waltham, MA) or Alexa 488-labeled rat anti-mouse CD11b (Abcam) or Alexa 647-conjugated mouse anti-NK1.1 antibody (Invitrogen) at 37°C for 1 hour. For pRIPK3 and pMLKL staining, samples were incubated with primary rabbit anti-mouse pRIPK3 antibody (Cell Signaling Technology, Danvers, MA) at 1:100 dilution in universal antibody dilution solution (Sigma Aldrich, St. Louis, MO) for 1 hour, then washed in PBS and incubated with Alexa 488-labeled goat anti-rabbit secondary antibody (Invitrogen, Waltham, MA). After washing, samples were stained with rabbit anti-mouse anti-pMLKL antibody (Cell Signaling Technology) directly conjugated to Alexa 555 using the Zenon Antibody Labeling Kit (Thermo Fisher, Waltham, MA) for 1 hour. For HMGB1, 4-HNE and CD8 staining, samples were incubated with mouse anti-HNEJ2 antibody (Abcam) and rabbit anti-mouse CD8a antibody (Abcam) at 4°C overnight. Samples were then washed and counterstained with Alexa 555-labeled goat anti-rabbit and Alexa 647 goat anti-mouse secondary antibodies (Invitrogen) at 37°C for 45 minutes. After washing, samples were incubated with mouse anti-HMGB1 antibody at 37°C for 1 hour. Samples were then washed and blocked in goat serum, incubated with Alexa 555-conjugated goat anti-rabbit secondary antibody (Invitrogen) for 45 minutes, and washed in PBS for 30 minutes. Samples were then incubated with rabbit anti-mouse HMGB1 antibody directly conjugated to Alexa 488 (Abcam) for 1 hour at 37°C. Samples were washed in PBS and quenched for autofluorescence and mounted as described (37). Samples were visualized at 10X or 20X magnification using a Keyence BZ-800 microscope (Keyence, Osaka, Japan) under 10X objective or 20X objective using a 1X digital zoom, and image capture was performed using a DXM1200F camera using software supplied by the vendor (Nikon). Spatial intensity of staining was performed in ImageJ, using phase contrast microscopy to identify the zone of histotripsy tumor ablation. Entire tumor sections were examined for immunohistochemical analysis, and fluorescence intensity of staining was calculated over every 100 μm^2^ area. A minimum of 5 fields of view from two independent experiments were considered for intensity analysis and statistical analysis. Images were imported to ImageJ and the threshold was adjusted using the color threshold tool. Cells were then counted using ImageJ software after appropriate background subtraction. Cells lacking intranuclear HMGB1 were scored as cells that had released HMGB1, and cells containing intranuclear HMGB1 were scored as cells that had retained HMGB1. Nuclear localization was confirmed by colocalization with DAPI. To measure the spatial correlation between CD8+ T cells and tumor cell HMGB1 release or 4-HNE accumulation, a region of interest of 50 μm diameter was drawn around CD8+ cells, and the percent of tumor cells without HMGB1 or with 4-HNE was calculated.

### RNA extraction, sequencing, and analysis

Flow-sorted CD45+ and CD45- cells were lysed and RNA was extracted per manufacturer instructions using the Qiagen RNeasy Mini Kit (Qiagen, Hilden, Germany). RNA was immediately frozen for storage and submitted to the University of Michigan Advanced Genomics Core for library preparation and QuantSeq analysis. Pooled libraries were subjected to 101 bp single-end sequencing with Illumina NovaSeq following manufacturer protocols (Illumina, San Diego, CA). Bcl2fastq2 Conversion Software (Illumina) was used to generate de-multiplexed Fastq files. Reads were trimmed using Trim Galore (v0.5.0) (https://www.bioinformatics.babraham.ac.uk/projects/trim_galore/) using default settings. Reads were aligned using STAR (v 2.6.0) using default settings (39). Unique molecular identifiers were collapsed using Lexogen Quantseq (v2.3.6). Reads were mapped to the mouse genome GRCm38 from the Ensembl genome database. Reads were counted using Bioconductor R package Rsubread and featureCounts function (40). RNASeq data analysis and visualizations were performed using BioJupies (41). Methods derived from the Biojupies analyses are summarized in the following passages. Raw counts were normalized to log10-Counts Per Million (logCPM) by dividing each column by the total sum of its counts, multiplying by 10^6^, and applying a log10-transform. Principal component analysis (PCA) was performed using the PCA function from the scikit-learn Python module. Before performing PCA, raw gene counts were normalized using the logCPM method, filtered by selecting the 500 genes with the most variable expression, and transformed using the Z-score method. Heatmaps were generated in Graphpad (GraphPad Software, San Diego, CA) by plotting the Z-scores for the top 2500 differentially-expressed genes associated with a relevant gene ontology (42–44).

### Digital spatial profiling

The Nanostring GeoMx Digital Spatial Profiling (DSP) (Nanostring, Seattle, WA) platform was used to quantify expression levels of immune-relevant proteins in specific areas of lymphocytic infiltration present within tumors (45). Tumor sections of 5 μm thickness were stained with fluorescent antibodies against CD8 and CD45 and S100B, and 12 regions of interest representing areas of lymphocytic infiltration measuring 200 μm in diameter were selected per tumor. Protein profiling data were provided by NanoString through their Technology Access Program.

### Flow cytometry

The following fluorophore-conjugated antibodies were used in this study: αCD45-Alexa Fluor 488 or -PerCP-Cy5.5 (30-F11), αCD8α-APC-Cy7 or Pacific Blue (53-6.7), αCD4-BB700 or -APC-Cy7 (RM4–5), αCD3/CD3ϵ-Brilliant violet^®^ (BV)605 (17A2 or 145-2C11), αCD11b-BV510 (M1/70), αF4/80-PE-Cy5 (BM8), αCD11c-PE-Cy7 (N418), αCD69-APC (H1.2F3), αLy6G-PE (1A8), αLy6C-PE-Dazzle 594 (HK1.4), αPD-1-PE (RMP1-14), αCD161-AF488 (694370), αCD25-PeCy5 (PC61), αCD19-PE-Cy7 (6D5), αCD127-BV510 (A7R34), αCD62L-AF647 (MEL-14), αCD44-PE-CF594 (IM7). (All antibodies were purchased from BioLegend (San Diego, CA), eBioscience (San Diego, CA), BD Biosciences (San Diego, CA), R&D Systems (Minneapolis, MN), or Cell Signaling Technology.) Cells were stained with a viability marker (Live/Dead Near-IR (Thermo Fisher) or DAPI according to manufacturer’s instructions and stained for surface markers following Fc receptor blocking with TruStain FcX (BioLegend) per manufacturer’s instructions. Immediate staining after tissue harvest was performed for sorting of CD45+ or CD45- populations; cells for analysis of surface marker expression with flow cytometry were initially frozen in RPMI-1640 with 10% FBS and 10% DMSO and thawed and stained on the day of analysis.

### Co-culture experiments

C57BL/6 mice bearing unilateral B16F10 flank tumors were treated with no therapy or histotripsy ablation on day 10. Mice were euthanized on day 15 and tumor-draining inguinal lymph nodes were harvested. CD8+ T cells were isolated from lymph nodes using the EasySep™ cell isolation system (STEMCELL). 10^6^ CD8+ T cells were added to cultured B16F10 cells in 1.5 mL tubes in RPMI-1640 with 10% FBS for 90 minutes at 37°C. Tubes were then centrifuged and plated on poly-lysine coated glass slides for 10 minutes in a humid chamber at 37°C to allow for adherence. Cells were fixed with 3.5% buffered parformaldehyde and permeablized with 0.5% Triton X-100 and blocked in 5% bovine serum albumin (Sigma). Cells were first stained overnight with αHNEJ2 antibody (1:100 dilution) (Abcam) at 4°C, then counterstained with goat anti-mouse IgG antibody (1:200 dilution) (Thermo Fisher) and stained with αCD8 antibody directly conjugated to Alexa 488 (1:100 dilution) (BioLegend). After staining, slides were washed in PBS, mounted with prolonged gold-containing DAPI, and visualized. Ten fields of view were captured under 20X magnification, and the number of 4-HNE-positive cells were counted relative to the total number of B16F10 cells.

### Vaccination studies

C57BL/6 mice bearing unilateral B16F10 flank tumors were treated with no therapy, histotripsy ablation, or 15 Gy external beam radiation therapy on day 10. Mice were euthanized on day 11 and tumors were explanted. Tumors that received no treatment were exposed to 3 cycles of rapid freezing for 2 minutes in liquid nitrogen and thawing for 2 minutes in a 60°C water bath. Tumor homogenates were centrifuged at 1K RPM for 5 minutes, and the cell-free supernatant was injected intraperitoneally into naïve C57BL/6 mice one day prior to flank injection with B16F10 tumors.

### Statistical analysis

Statistical analysis was performed using GraphPad Prism software (GraphPad Software). The difference between means of unpaired samples was performed using two-way analysis of variance (ANOVA) with Bonferroni’s post-test or an unpaired t-test as indicated. Tumor growth kinetics were compared using ANOVA. Statistical significance was defined as p<0.05. Numbers of mice per experiment are noted in the Figure Legends.

### Study approval

All animal studies were performed in accordance with protocols and animal care and use guidelines approved by the Institutional Animal Care and Use Committees of the VA Ann Arbor Healthcare System and University of Michigan.

## Results

### Partial histotripsy ablation inhibits tumor growth rate in treated tumors and in distant untreated tumors

**Figure 1:**
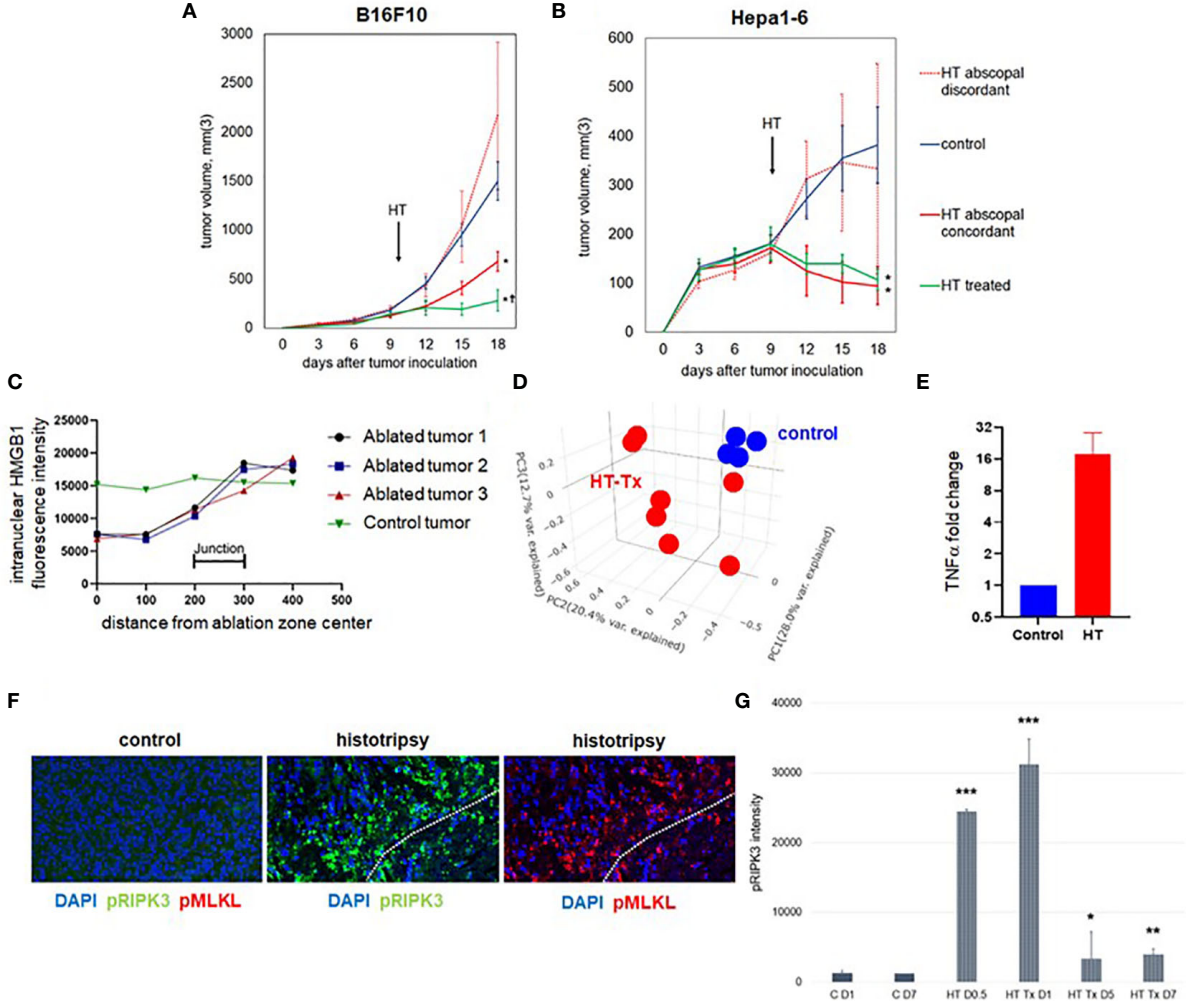
Unilateral histotripsy tumor ablation induces antigen-specific abscopal inhibition of distant, untreated tumors and local immunogenic cell death. **(A)** C57BL/6 mice were inoculated with bilateral flank B16F10 tumors (“HT abscopal concordant”) or unilateral B16F10 flank tumors and contralateral Hepa1-6 flank tumors (“HT abscopal discordant”), and sham (“control”) or histotripsy ablation encompassing ~80-90% of unilateral B16F10 flank tumors (in control and abscopal concordant groups) or unilateral Hepa1-6 flank tumors (in the abscopal discordant group) was performed on day 10. In contrast to sham ablation controls, mice treated with unilateral partial histotripsy ablation demonstrated immediate local growth arrest of treated B16F10 tumors (“HT treated”) and immediate abscopal growth inhibition of distant untreated B16F10 tumors (“HT abscopal concordant”) but not distant untreated B16F10 tumors after contralateral Hepa1-6 tumor ablation (“HT abscopal discordant”). **(B)** In mice bearing bilateral flank Hepa1-6 tumors or unilateral Hepa1-6 flank tumors and contralateral B16F10 flank tumors, unilateral partial histotripsy ablation of Hepa1-6 tumors (in control and HT abscopal concordant groups) or B16F10 tumors (in the HT abscopal discordant group) demonstrated immediate growth arrest and regression of treated Hepa1-6 tumors (“HT treated”) and distant untreated Hepa1-6 tumors (“HT abscopal concordant”), but not of distant untreated Hepa1-6 tumors after contralateral B16F10 tumor ablation (“HT abscopal discordant”). **(C)** Multicolor immunofluorescence analysis of bilateral B16F10 tumors performed 2 days after unilateral sham or partial histotripsy ablation demonstrated homogeneous intranuclear staining of HMGB1 in control tumors. In contrast, significant loss of intranuclear HMGB1 staining was observed within the ablation zone of histotripsy-treated tumors; intranuclear HMGB1 was retained outside of the ablation zone. **(D)** RNASeq of CD45- tumor cells performed on day 13 revealed marked differences in transcriptional activity between control tumors (blue) and histotripsy-treated (“HT-Tx”) tumors (red) as evidenced by principal component analysis. **(E)** qRT-PCR of tumors performed on day 13 revealed an approximately 20-fold increase in TNFα mRNA in histotripsy-treated tumors (red) compared with control tumors (blue). **(F)** Multicolor immunohistochemistry 1 day after sham or histotripsy ablation revealed no measurable expression of the necroptosis markers pRIPK3 and pMLKL in control tumors; in contrast, profound co-localized expression of pRIPK3 and pMLKL was seen along the periphery of ablated zones in histotripsy-treated tumors. **(G)** Serial quantitation of pRIPK3 fluorescence intensity in control (“C”) and histotripsy-treated (“HT-Tx”) tumors over various time points demonstrated rapid and transient upregulation of necroptosis-associated phosphorylated protein levels following histotripsy ablation. [**(A, B):** n=7-9 mice per group; *=p<0.05 compared with control tumors; †=p<0.05 compared with HT abscopal concordant tumors. **(C-G):** n=3-4 mice per group; *=p<0.05 compared with control day 1 tumors; **=p < 0.01 compared with control day 1 tumors; ***=p < 0.0001 compared with control day 1 tumors].

To compare the effects of histotripsy on tumors treated with partial histotripsy ablation (“histotripsy-treated” tumors) and distant untreated (“histotripsy-abscopal”) tumors, as well as across tumors of different histology and immunogenicity, we performed unilateral sham (control) versus partial (~80-90% tumor volume) histotripsy tumor ablations in C57BL/6 mice bearing bilateral flank B16F10 melanoma or Hepa1-6 hepatocellular carcinoma tumors. Partial tumor ablations were performed in order to avoid collateral damage to surrounding subcutaneous tissues. Whereas poorly immunogenic B16F10 tumors exhibit exponential growth, moderately immunogenic Hepa1-6 tumors exhibit linear growth. Ablations were performed on days 9-10 after tumor inoculation, when tumor volumes exceeded 7 mm in diameter. As shown in **Figures 1A, B**, partial histotripsy ablation caused immediate growth arrest of histotripsy-treated B16F10 tumors and gradual regression of histotripsy-treated Hepa1-6 tumors. As previously reported (37), unilateral histotripsy was accompanied by abscopal inhibition of contralateral histotripsy-abscopal B16F10 tumors. In the Hepa1-6 tumor model, unilateral histotripsy was accompanied by growth arrest and gradual regression of histotripsy-abscopal tumors. This abscopal effect appeared antigen-specific, as no abscopal growth inhibition was observed in contralateral tumors of discordant pathology. These observations confirm that unilateral histotripsy tumor ablation results in rapid abscopal growth inhibition of contralateral, non-ablated tumors across two different histological tumor types.

### Partial histotripsy ablation is followed by early immunogenic cell death

As previously reported (37), multicolor immunohistochemistry revealed significant loss of intranuclear HMGB1 within the ablation zone on days 1-3 after partial histotripsy ablation (**Figure 1C**). Despite partial ablation in which portions of histotripsy-treated tumors were left intact and unaffected by histotripsy, global RNASeq of CD45- tumor cells performed 3 days after sham or histotripsy ablation revealed significant differences in transcriptional activity following partial histotripsy (**Figure 1D**). Gene ontology enrichment analysis (**Supplemental Figure 1**) revealed that the most substantial increases in transcriptional activity in tumor cells following histotripsy were among pathways relevant to “cellular response to heat” (Z-score 12.2, FDR 3.9x10^-5^, p=1.7x10^-8^) and “response to unfolded protein” (Z-score 12.0, FDR=9.3x10^-5^, p=8x10^-8^); the most substantial decreases in transcriptional activity following histotripsy was among pathways relevant to “regulation of transcription from RNA polymerase II promoter” (Z-score 1.84, FDR=0.051, p=2.2x10^-5^). These pathway changes are consistent with responses to severe cellular and endoplasmic reticulum (ER) stress. Quantitative real-time polymerase chain reaction (qRT-PCR) demonstrated significant increases in TNFα transcription within treated tumors 3 days after histotripsy (**Figure 1E**). Immunohistochemical analysis of morphologically intact tumor cells at the junction of histotripsy ablated and non-ablated zones demonstrated rapid and transient colocalized upregulation of pRIPK3 and pMLKL (**Figures 1F, G** **)** indicating the onset of necroptosis, a TNFα-driven pathway of immunogenic cell death (46, 47).

**Figure 2:**
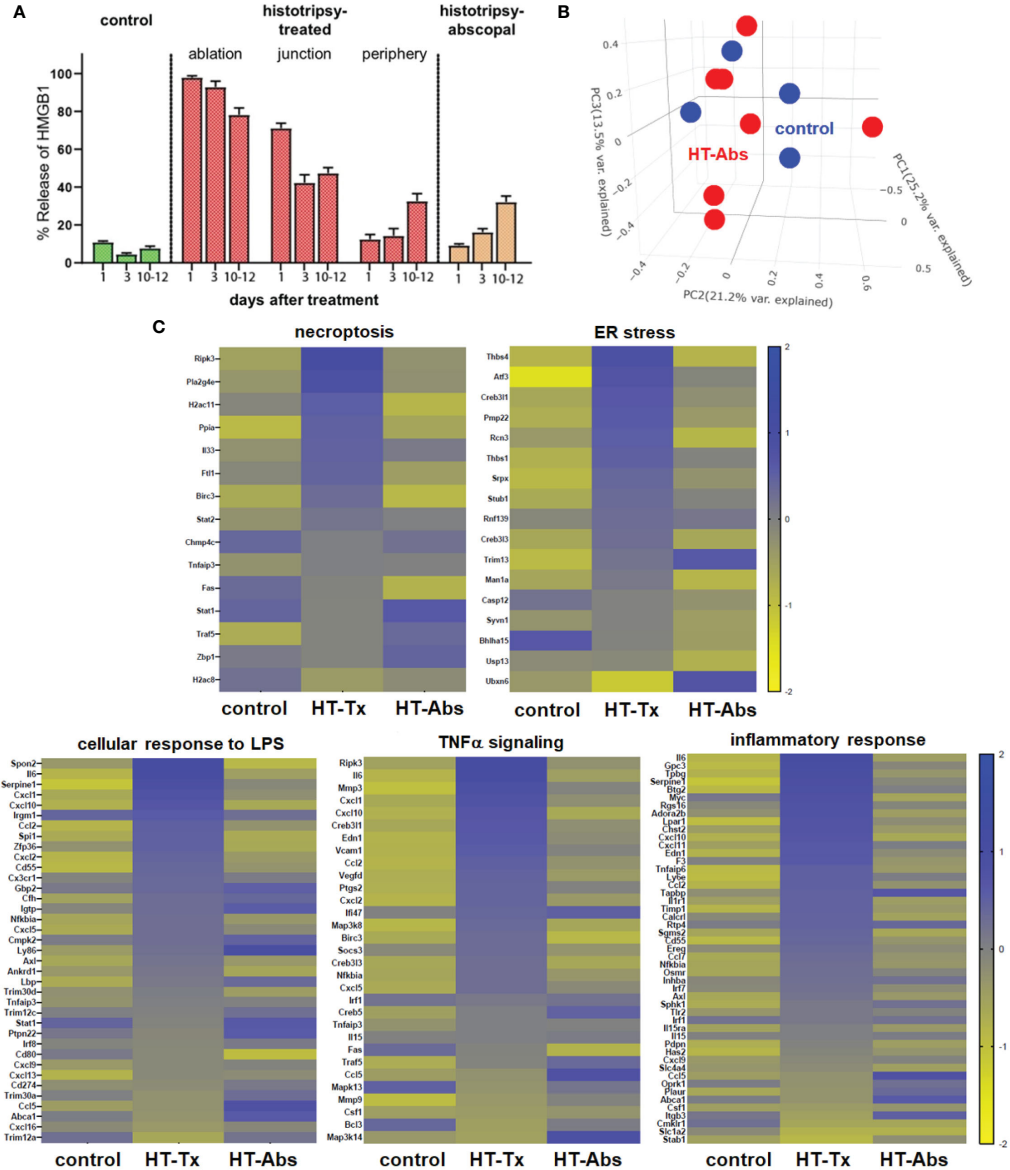
Early cellular stress and immunogenic cell death is observed in treated tumors but not distant untreated tumors following histotripsy. Mice bearing bilateral B16F10 tumors were treated with unilateral sham (control) or partial histotripsy ablation on day 10. **(A)** Multicolor immunofluorescence microscopy analysis of control, histotripsy-treated, and histotripsy-abscopal tumors on days 1, 3 or 10-12 after unilateral sham or partial histotripsy ablation revealed no significant release of intranuclear HMGB1 in control tumors at any time point. Histotripsy-treated tumors exhibited significant and immediate loss of nuclear HMGB1 staining that was highest within the ablation zone and the junction of ablated and peripheral non-ablated zones at early time points. At later time points, progressive loss of nuclear HMGB1 was observed within peripheral non-ablated zones of histotripsy-treated tumors. Similarly, whereas contralateral histotripsy-abscopal tumors demonstrated no HMGB1 translocation at early time points, progressive loss of intranuclear HMGB1 staining was observed at later time points. **(B)** RNASeq of CD45- tumor cells from sham-treated control tumors (blue) and histotripsy-abscopal (“HT-Abs”) tumors (red) performed 3 days after unilateral partial histotripsy revealed no substantial differences in overall mRNA transcriptional activity as evidenced by principal component analysis. **(C)** RNASeq of CD45- tumor cells 3 days after sham or unilateral histotripsy ablation demonstrated upregulated transcription of genes associated with necroptosis, ER stress, cellular response to LPS, TNFα signaling and inflammatory response in histotripsy-treated tumors (“HT-Tx”) as compared to control and histotripsy-abscopal (“HT-Abs”) tumors. (n=3-5 mice per experimental group).

In contrast to histotripsy-treated tumors, histotripsy-abscopal tumors exhibited no measurable extranuclear translocation of HMGB1 immediately after contralateral histotripsy ablation (**Figure 2A**). Moreover, RNASeq of CD45- tumor cell populations performed 3 days after sham or histotripsy ablation revealed no clear differences in overall patterns of transcriptional activity between control tumors and histotripsy-abscopal tumors (**Figure 2B**); in contrast to control tumors, upregulated transcriptional activity of genes associated with necroptosis, ER stress, cellular response to LPS, TNFα signaling, and inflammatory responses were observed in histotripsy-treated tumors but not histotripsy-abscopal tumors (**Figure 2C**).

### Partial histotripsy ablation triggers an early local inflammatory response

**Figure 3:**
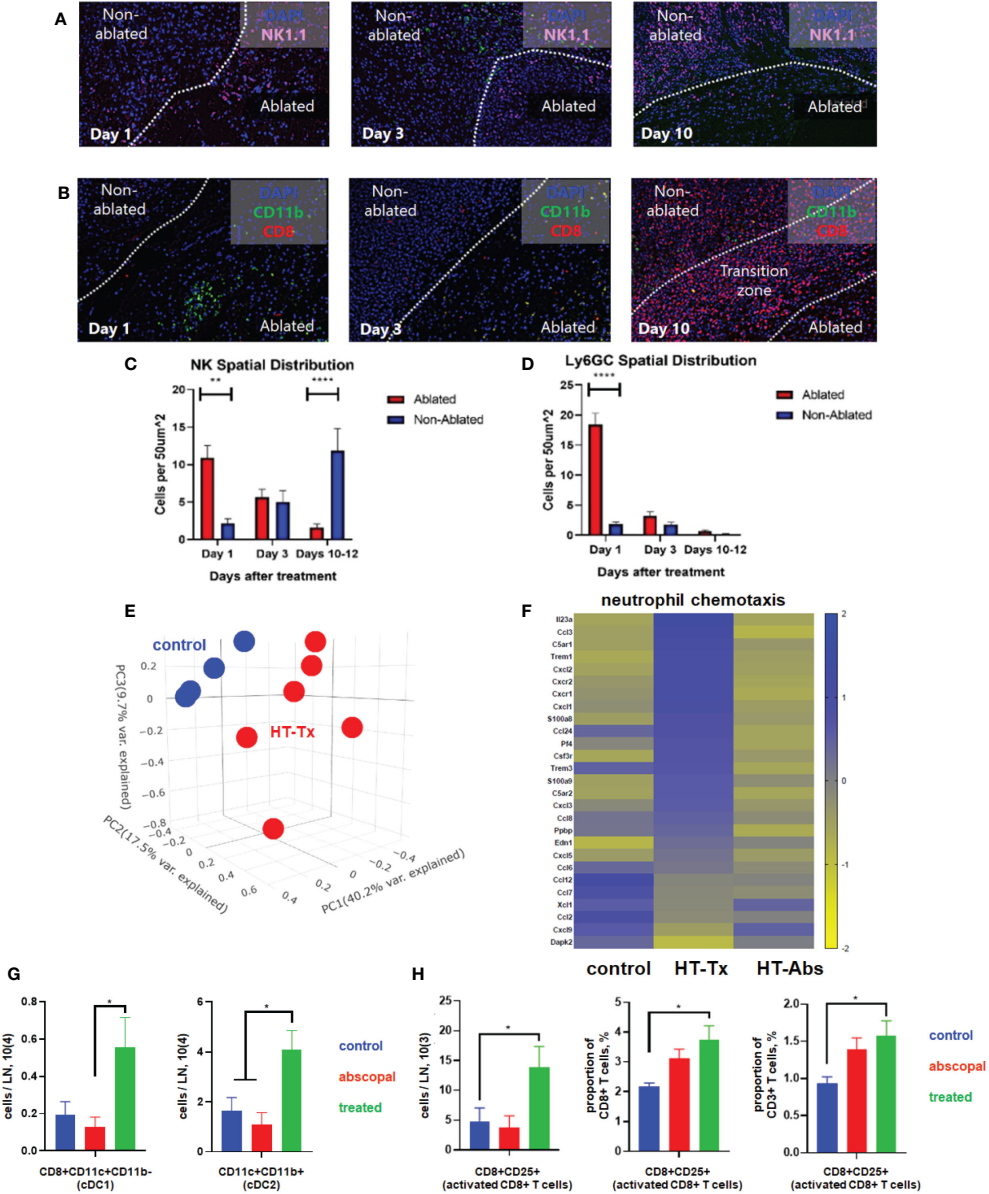
Histotripsy ablation is followed by infiltration of innate and adaptive immune cell populations. Mice bearing bilateral B16F10 tumors were treated with unilateral sham (control) or partial histotripsy ablation on day 10. Multicolor immunofluorescence performed 1, 3 and 10 days after sham or histotripsy partial histotripsy ablation revealed **(A)** early infiltration of NK1.1+ cells initially localized within the histotripsy ablated zone that gradually migrated outward toward peripheral, non-ablated zones on days 3 and 10, and **(B)** early and transient infiltration of CD11b+ cells within the histotripsy ablated zone on day 1 and delayed infiltration of CD8+ T cells within the non-ablated zone on day 10. **(C)** Quantitation of NK1.1+ cells within ablated zones (red) and non-ablated zones (blue) of histotripsy-treated tumors at various time points revealed gradual migration of NK cells away from ablated zones toward non-ablated zones. **(D)** Quantitation of Ly6GC+ cells at various time points after histotripsy confirmed immediate but short-lived infiltration strictly localized to the ablated zone. **(E)** RNASeq of CD45+ immune cells performed 3 days after unilateral partial histotripsy ablation revealed marked differences in transcriptional activity between control tumors (blue) and histotripsy-treated (“HT-Tx”) tumors (red) as evidenced by principal component analysis. **(F)** Upregulated transcription of genes associated with neutrophil chemotaxis was observed in histotripsy-treated but not histotripsy-abscopal (“HT-Abs”) tumors. **(G)** Mice bearing bilateral Hepa1-6 tumors were treated with unilateral partial histotripsy ablation on day 10. Flow cytometric analysis of tumor-draining lymph nodes of control (blue), histotripsy-treated (green) and histotripsy-abscopal (red) tumors performed on day 13 revealed significant increases in cDC1 and cDC2 populations within lymph nodes draining histotripsy-treated tumors. **(H)** Similarly, flow cytometric analysis revealed significant increases in activated CD8+ T cells within lymph nodes draining histotripsy-treated tumors. (n=3-6 mice per group; *=p<0.05 between groups; **=p < 0.01 between groups; ****=p < 0.0001 between groups).

Concordant with our previously-published flow cytometric findings (37), multicolor immunohistochemistry 3 days after histotripsy ablation revealed significant infiltration of NK cell and CD11b+ myeloid cell populations within the ablation zone (**Figures 3A, B** **)**. Time course experiments revealed that CD11b+ and Ly6GC+ myeloid cell infiltration was short-lived and confined to the tumor ablation zone, whereas NK1.1+ cells exhibited a gradual centrifugal pattern of outward infiltration away from the ablation zone and into peripheral non-ablated zones (**Figures 3A–D**).

RNASeq of CD45+ immune cell populations 3 days after sham or histotripsy ablation revealed significant differences in transcriptional activity following histotripsy (**Figure 3E**). The most substantial increases in transcriptional activity of immune cells were among pathways relevant to “response to molecule of bacterial origin” (Z-score 6.1, FDR=0.0018, p=9.9x10^-7^) and “response to lipopolysaccharide” (Z-score 4.6, FDR=0.0018, p=1.8x10^-6^) (**Supplemental Figure 2**), consistent with an intense innate immune response to inflammation. Upregulated transcriptional activity of genes associated with neutrophil chemotaxis was observed in histotripsy-treated tumors but not control and histotripsy-abscopal tumors (**Figure 3F**).

In order to evaluate priming of adaptive immune responses following histotripsy, flow cytometric analysis of tumor-draining lymph nodes 3 days after sham or histotripsy ablation revealed significant increases in numbers of conventional type 1 and type 2 dendritic cells (cDC1 and cDC2) within lymph nodes draining histotripsy-treated tumors (**Figure 3G**). Concurrently, significant increases in activated phenotype were observed among CD8+ T cells within histotripsy-treated tumor-draining lymph nodes (**Figure 3H**), suggesting enhanced APC:T cell priming conditions.

### Partial histotripsy ablation triggers gradual infiltration of CD8+ T cells into non-ablated tumor zones

**Figure 4:**
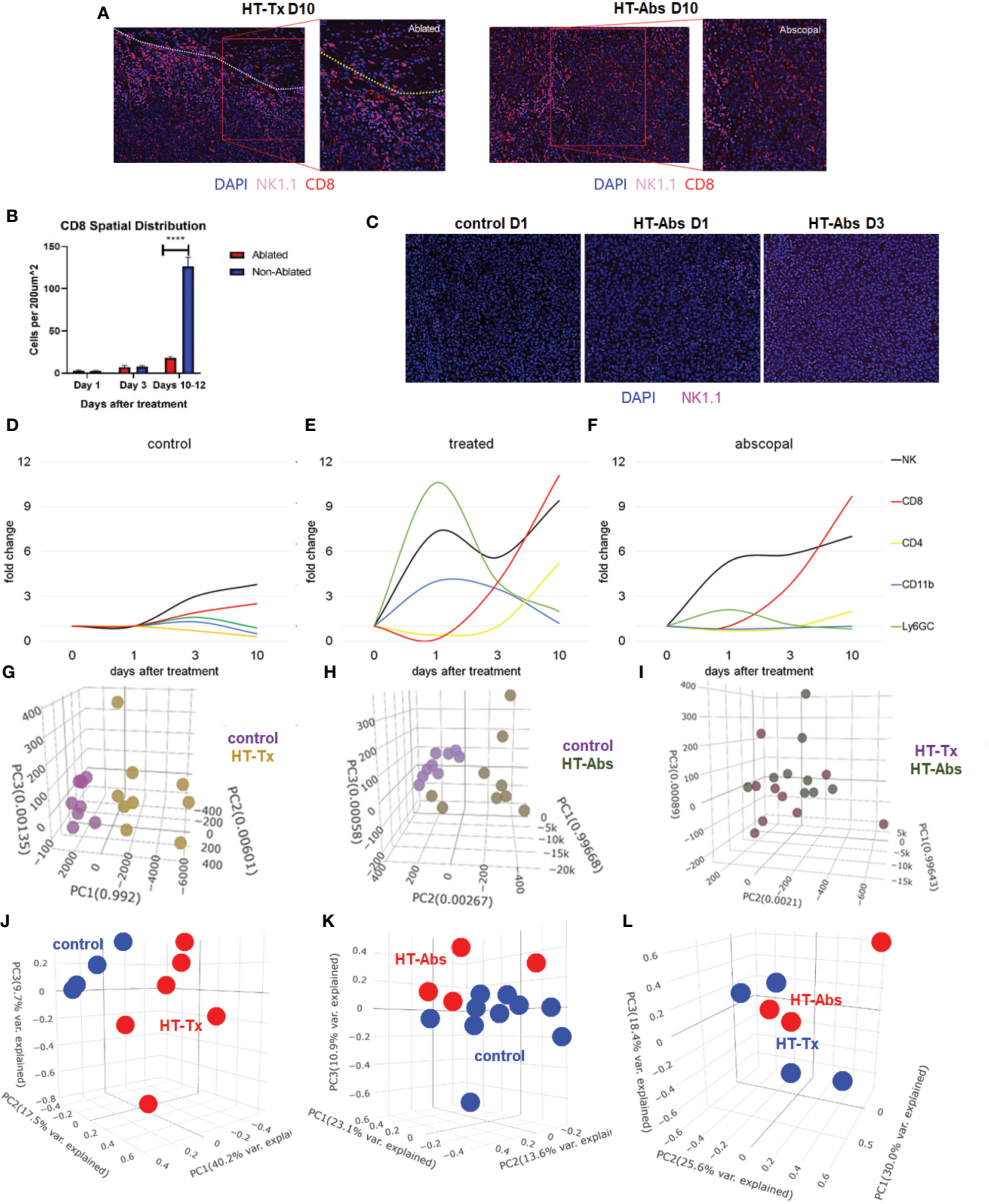
The temporal kinetics of intratumoral cell infiltration are dissimilar at early time points and similar at later time points between histotripsy-treated and histotripsy-abscopal tumors. Mice bearing bilateral B16F10 tumors were treated with unilateral sham (control) or partial histotripsy ablation on day 10. **(A)** Multicolor immunofluorescence 10 days after unilateral B16F10 histotripsy ablation showed an influx of NK1.1+ and CD8+ cell populations within the non-ablated zones of histotripsy-treated (“HT-Tx”) tumors and diffusely in histotripsy-abscopal (“HT-Abs”) tumors. **(B)** Quantitation of CD8+ staining at various time points revealed delayed infiltration that was strictly localized to non-ablated zones (blue) and not ablated zones (red). **(C)** Multicolor immunofluorescence at early time points revealed infiltration of NK1.1+ cells in histotripsy-abscopal (“HT-Abs”) tumors. **(D-F)** Multicolor immunohistochemistry performed 1, 3 and 10 days after partial histotripsy ablation revealed minimal increases in NK1.1+ and CD8+ cells in control tumors over time **(D)**. In contrast, histotripsy-treated tumors exhibited rapid influx of CD11b+ and Ly6GC+ and NK1.1+ cell populations immediately after ablation; whereas CD11b+ and Ly6CG+ cell infiltration was short-lived, NK1.1+ cell populations followed a biphasic pattern of early and delayed intratumoral infiltration; CD8+ and CD4+ cell infiltration followed a delayed pattern of delayed intratumoral infiltration **(E)**. Whereas histotripsy-abscopal tumors did not exhibit the early pattern of CD11b+ and Ly6GC+ cell infiltration seen in histotripsy-treated tumors, patterns of NK1.1+ and CD8+ and CD4+ cell populations were similar to those observed in histotripsy-treated tumors **(F)**. **(G-I)** Digital spatial profiling of immune-relevant protein expression within regions of lymphocytic infiltration was performed on control, histotripsy-treated (“HT-Tx”) and histotripsy-abscopal (“HT-Abs”) B16F10 tumors 10 days after unilateral sham or histotripsy ablation. Principal component analyses demonstrated differences in overall expression of immune-relevant proteins between control (purple) and histotripsy-treated (gold) tumors **(G)**, and between control (purple) and histotripsy-abscopal (bronze) tumors **(H)**; however, patterns of immune-relevant protein expression were largely superimposable between histotripsy-treated (brown) and histotripsy-abscopal (green) tumors **(I)**. **(J-L)** RNASeq analyses of intratumoral CD45+ cells were performed on control, histotripsy-treated (“HT-Tx”) and histotripsy-abscopal (“HT-Abs”) B16F10 tumors 10 days after unilateral sham or histotripsy ablation. Principal component analyses demonstrated differences in overall transcriptional activity between control (blue) and histotripsy-treated (red) tumors **(J)** and between control (red) and histotripsy-abscopal tumors (blue) **(K)**, but not between histotripsy-treated (blue) tumors and histotripsy-abscopal (red) tumors **(L)**. (n=3-10 mice per experimental group; ****=p < 0.0001 between groups).

Also concordant with our previously-published flow cytometric findings (37), multicolor immunohistochemistry revealed delayed infiltration of CD8+ T cells 10 days after histotripsy. This infiltration was spatially confined to non-ablated tumor zones of histotripsy-treated tumors, with no appreciable CD8+ T cell infiltration observed within ablation zones (**Figures 4A, B** **)**. As had been seen with flow cytometric analyses (24), examination of contralateral histotripsy-abscopal tumors on day 10 revealed diffuse infiltration of CD8+ T cells into distant tumor sites (**Figures 4A, B** **)**. These findings suggest that early immunogenic inflammatory responses and APC mobilization after histotripsy may enable subsequent homing of tumor-reactive CD8+ T cells into non-ablated tumor sites.

### Similarities and differences between local and distant abscopal responses to histotripsy

Similar to histotripsy-treated tumors, early infiltration of NK cells was observed in histotripsy-abscopal tumors (**Figure 4C**). However, the overall temporal kinetics of local and abscopal intratumoral cell infiltration were qualitatively different at early time points, with histotripsy-treated tumors exhibiting a rapid early inflammatory response of transient myeloid cell infiltration that was absent in histotripsy-abscopal tumors (**Figures 4D–F**). Local and abscopal responses were both characterized by delayed infiltration of NK cells and CD8+ and CD4+ T cells. The parallels in immune phenotype observed at later time points between treated and abscopal tumors were also reflected in digital spatial profiling (DSP) of immune-relevant protein expression. DSP of protein expression within areas of lymphocytic infiltration in control, histotripsy-treated and histotripsy-abscopal B16F10 tumors 10 days after sham or histotripsy ablation revealed stark differences between control tumors and histotripsy-treated tumors (**Figure 4G**) and between control tumors and histotripsy-abscopal tumors (**Figure 4H**). However, protein expression profiles were largely superimposable between histotripsy-treated tumors and histotripsy-abscopal tumors (**Figure 4I**), suggesting that the delayed anti-tumor adaptive immune responses following histotripsy ablation are global and systemic in nature. Proteins with significant differences in expression levels between groups were involved in optimal T cell function and antigen presentation (**Table 1**). Similarly, RNASeq analysis of CD45+ immune cell populations 7 days after sham or histotripsy tumor ablation revealed diverging patterns of transcriptional activity between control tumors and histotripsy-treated tumors (**Figure 4J**) and between control and histotripsy-abscopal tumors (**Figure 4K**) but not between histotripsy-treated and histotripsy-abscopal tumors (**Figure 4L**). Transcriptional activity was most substantially upregulated in pathways associated with “inflammatory response” (Z-score 3.27, FDR=0.038, p=2.1x10^-5^) in histotripsy-treated tumors (**Supplemental Figure 3**) and “cellular response to type I interferon” (Z-score 15.5, FDR=2.3x10^-11^, p=1.8x10^-14^) in histotripsy-abscopal tumors (**Supplemental Figure 4**). Moreover, the most substantial transcriptional differences between histotripsy-treated and histotripsy-abscopal tumors were upregulated pathways of innate immune response including “inflammatory response” (Z-score 4.26, FDR=5.4x10^-5^, p=2.3x10^-8^) and “regulation of neutrophil chemotaxis” (Z-score 15.1, FDR=4.5x10^-4^, p=3.9x10^-7^) in histotripsy-treated but not histotripsy-abscopal tumors (**Supplemental Figure 5**). These findings indicate that, whereas the early effects of histotripsy are characterized by stronger inflammatory and innate immune responses in histotripsy-treated tumors, the delayed effects of histotripsy are characterized by similar local and abscopal enhanced adaptive immune responses.

**Table 1:** Differential expression of immune-relevant proteins between control, histotripsy-treated, and histotripsy-abscopal tumors on day 7.

HT treated vs. control	HT abscopal vs. control	HT treated vs. abscopal
B7H3	F4/80	GITR
F4/80	CD14	
IFNγR	CD45	
CD44	CD27	
fibronectin	CD40	
CD45	CD28	
CD27	IFNγR	
CD4	granzyme B	
CD40L	B7H3	
	CD4	
	GITR	
	CD11c	

### The abscopal effect of histotripsy is associated with ferroptotic cancer cell death co-localized with CD8+ T cells

**Figure 5:**
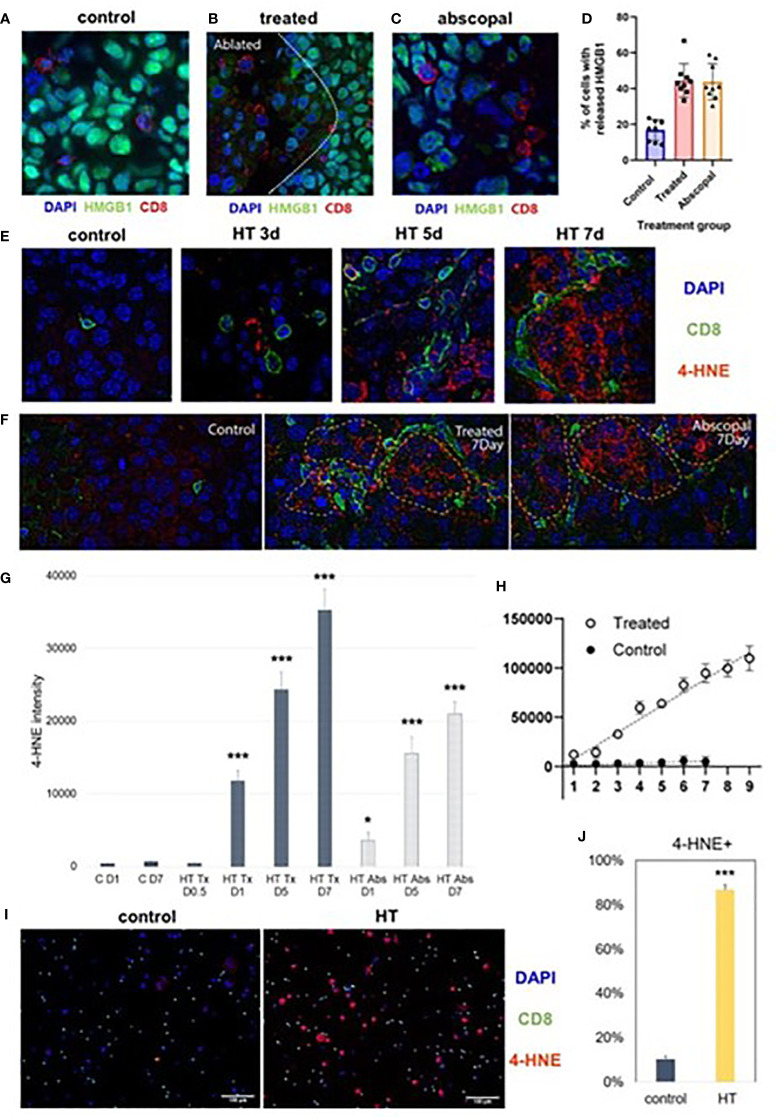
Late intratumoral CD8+ cell infiltration co-localizes with immunogenic and ferroptotic cancer cell death following histotripsy. Mice bearing bilateral B16F10 tumors were treated with unilateral sham (control) or partial histotripsy ablation on day 10. **(A)** Multicolor immunofluorescence performed on day 7 after sham or histotripsy ablation revealed minimal CD8+ cell infiltration and minimal extranuclear translocation of HMGB1 among cancer cells in control tumors. CD8+ cell infiltration was co-localized with loss of intranuclear HMGB1 staining in histotripsy-treated tumors **(B)** and in histotripsy-abscopal tumors **(C)**. **(D)** Significantly higher percentages of cancer cells located within 50 μm of a CD8+ cell exhibited loss of nuclear HMGB1 in histotripsy-treated and histotripsy-abscopal tumors as compared with controls. **(E)** Multicolor immunofluorescence performed on days 3, 5 and 7 after sham or histotripsy ablation revealed spatial co-localization of CD8+ cell infiltration with cancer cell accumulation of 4-HNE, a byproduct of ferroptosis, in histotripsy-treated tumors but not in control tumors. **(F)** Strong co-localization was observed between CD8+ cell infiltration and 4-HNE on day 7 in histotripsy-treated and histotripsy-abscopal tumors, but not in control tumors. **(G)** Gradual accumulation of 4-HNE staining intensity was observed in histotripsy-treated (“HT Tx”) and histotripsy-abscopal (“HT Abs”) tumor cells. **(H)** A linear relationship was observed between number of CD8+ cells present (x axis) and intensity of 4-HNE expression within 50 μm (y-axis) in histotripsy-treated tumors but not in control tumors. **(I, J)** Mice bearing B16F10 flank tumors were treated with sham or histotripsy tumor ablation on day 10, and tumor-draining lymph nodes were harvested on day 15. CD8+ T cells derived from tumor-draining lymph nodes were co-cultured with B16F10 melanoma cells *in vitro*. Significant accumulation of 4-HNE was observed within B16F10 melanoma cells co-cultured with CD8+ T cells derived from lymph nodes draining histotripsy-treated but not sham-treated tumors. (n=3-6 mice per group; *=p < 0.05 compared with control day 1 tumors; ***=p < 0.001 compared with control day 1 tumors).

Multicolor immunohistochemistry analyses revealed that non-ablated zones of histotripsy-treated tumors and histotripsy-abscopal tumors both exhibited gradual increases in extranuclear HMGB1 translocation at later time points after histotripsy ablation (**Figure 2A**). This delayed HMGB1 release following histotripsy coincided spatially with areas of CD8+ T cell infiltration (**Figures 5A–D**). In addition, focal accumulation of the ferroptotic cell death byproduct 4-HNE (34–36) was observed in areas of CD8+ T cell infiltration within non-ablated zones of histotripsy-treated tumors (**Figures 5E–G**) and within histotripsy-abscopal tumors (**Figures 5F, G** **)** at later time points. A strong spatial correlation was observed between CD8+ T cell infiltration and 4-HNE accumulation in histotripsy-treated and histotripsy-abscopal tumors, but not in untreated control tumors (**Figure 5H**). Under *in vitro* co-culture conditions, B16F10 melanoma cells exhibited significant accumulation of 4-HNE when mixed with CD8+ T cells derived from lymph nodes draining histotripsy-treated B16F10 tumors; in contrast, CD8+ T cells derived from untreated B16F10 tumor-draining lymph nodes mediated no measurable ferroptosis (**Figures 5I, J** **)**. These data suggest that the local and abscopal effects of histotripsy could be mediated by infiltration of CD8+ T cells capable of inducing ferroptotic immunogenic cancer cell death.

### Combination treatment with histotripsy plus checkpoint inhibition results in maximal ferroptosis

**Figure 6:**
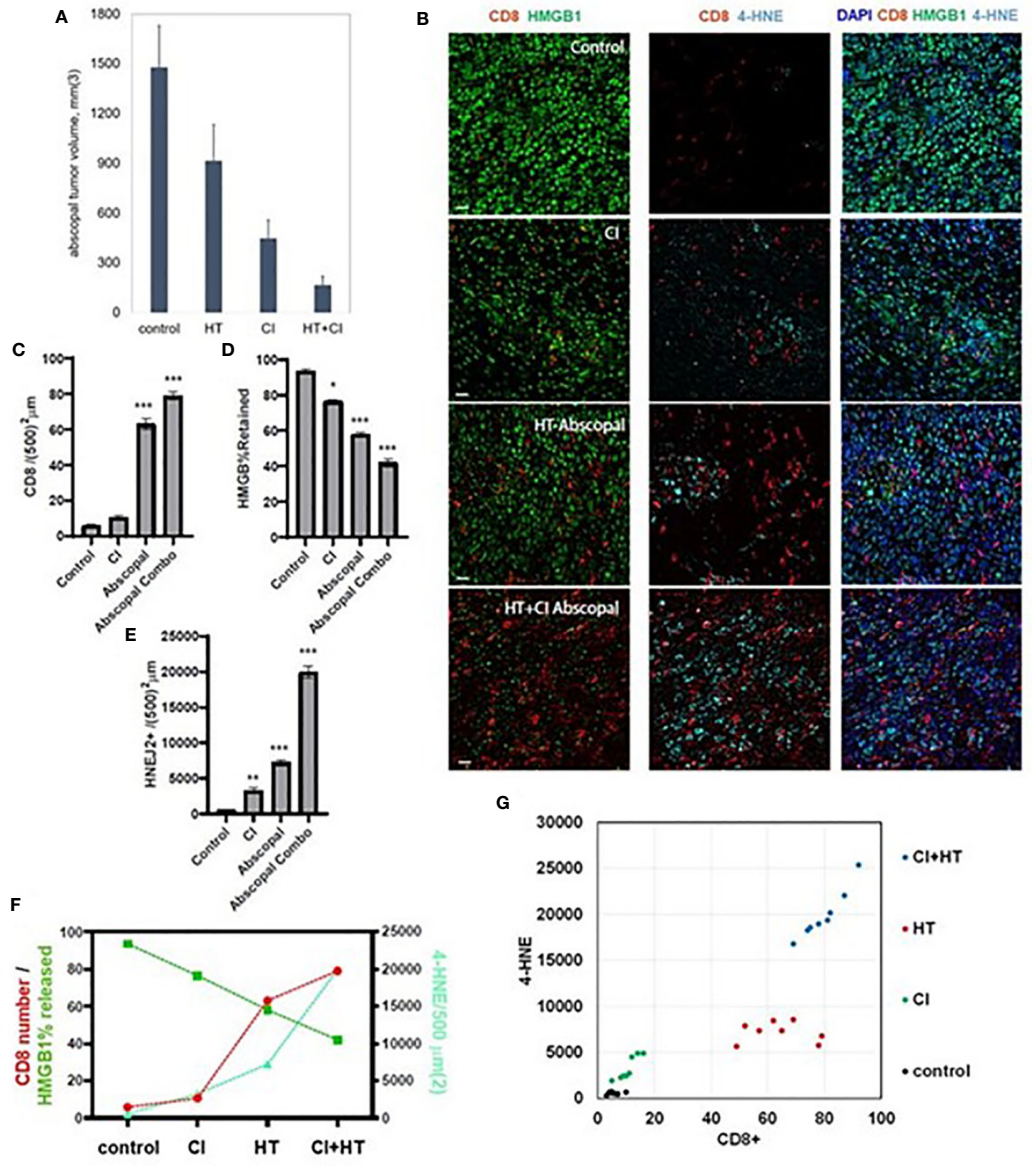
The combination of histotripsy with checkpoint inhibition results in additive intratumoral infiltration of CD8+ cells and synergistic induction of cancer cell ferroptosis. Mice bearing bilateral B16F10 tumors were treated with no therapy (control), checkpoint inhibition (CI) with anti-CTLA-4 mAb on days 6, 9 and 12 (“CI”), unilateral partial histotripsy ablation (“HT”) on day 7, or both (“HT+CI”). **(A)** Non-ablated abscopal tumor growth on day 18 was suppressed in mice treated with contralateral HT and CI, but maximal in mice treated with both. **(B)** Multicolor immunofluorescence of non-ablated abscopal tumors revealed increases in intratumoral CD8+ cell infiltration, loss of nuclear HMGB1, and 4-HNE accumulation after both CI and HT, with maximal effects seen after combinatorial HT+CI. **(C)** Quantitation of CD8+ cell density demonstrated an additive effect between CI and the abscopal effect of HT. **(D)** A similar additive effect was observed between CI and the abscopal effect of HT in extranuclear HMGB1 translocation. **(E)** The combination of HT+CI appeared to be a greater than additive effect on abscopal 4-HNE expression. The additive effects of CI and HT on abscopal CD8+ cell infiltration, HMGB1 release, and 4-HNE expression are shown in line graph form **(F)** and dot plot form **(G)**. (n=4-7 mice per group; *=p<0.05 compared with controls; **=p<0.01 compared with controls; ***=p<0.001 compared with controls).

Consistent with our previously published observations (37), the combination of histotripsy with anti-CTLA-4 checkpoint inhibition resulted in maximal abscopal control of non-ablated B16 and Hepa1-6 tumors (**Figure 6A**). It has recently been shown that the cytotoxic effects of CD8+ T cells primed by checkpoint inhibition are mediated by an acquired ability to induce cancer cell ferroptosis (18, 20). As shown in **Figure 6B**, multicolor immunohistochemistry analyses demonstrated that checkpoint inhibition and histotripsy both resulted in increased CD8+ T cell infiltration (**Figure 6C**), extranuclear translocation of HMGB1 (**Figure 6D**), and 4-HNE accumulation (**Figure 6E**). Abscopal CD8+ T cell infiltration and cancer cell HMGB1 release and 4-HNE accumulation were significantly greater after histotripsy than checkpoint inhibition. In addition, whereas the combination of histotripsy with checkpoint inhibition appeared to have additive effects on CD8+ T cell infiltration and HMGB1 release in abscopal tumors, combination therapy appeared to exert a synergistic induction of ferroptotic cancer cell death as measured by 4-HNE accumulation (**Figures 6F, G** **)**. These observations suggest that the therapeutic efficacy of combining histotripsy with checkpoint inhibition may be associated with their combined ability to promote CD8+ T cell-driven cancer cell ferroptosis.

### Tumor homogenates generated by histotripsy are immunogenic and have vaccine-like properties

**Figure 7:**
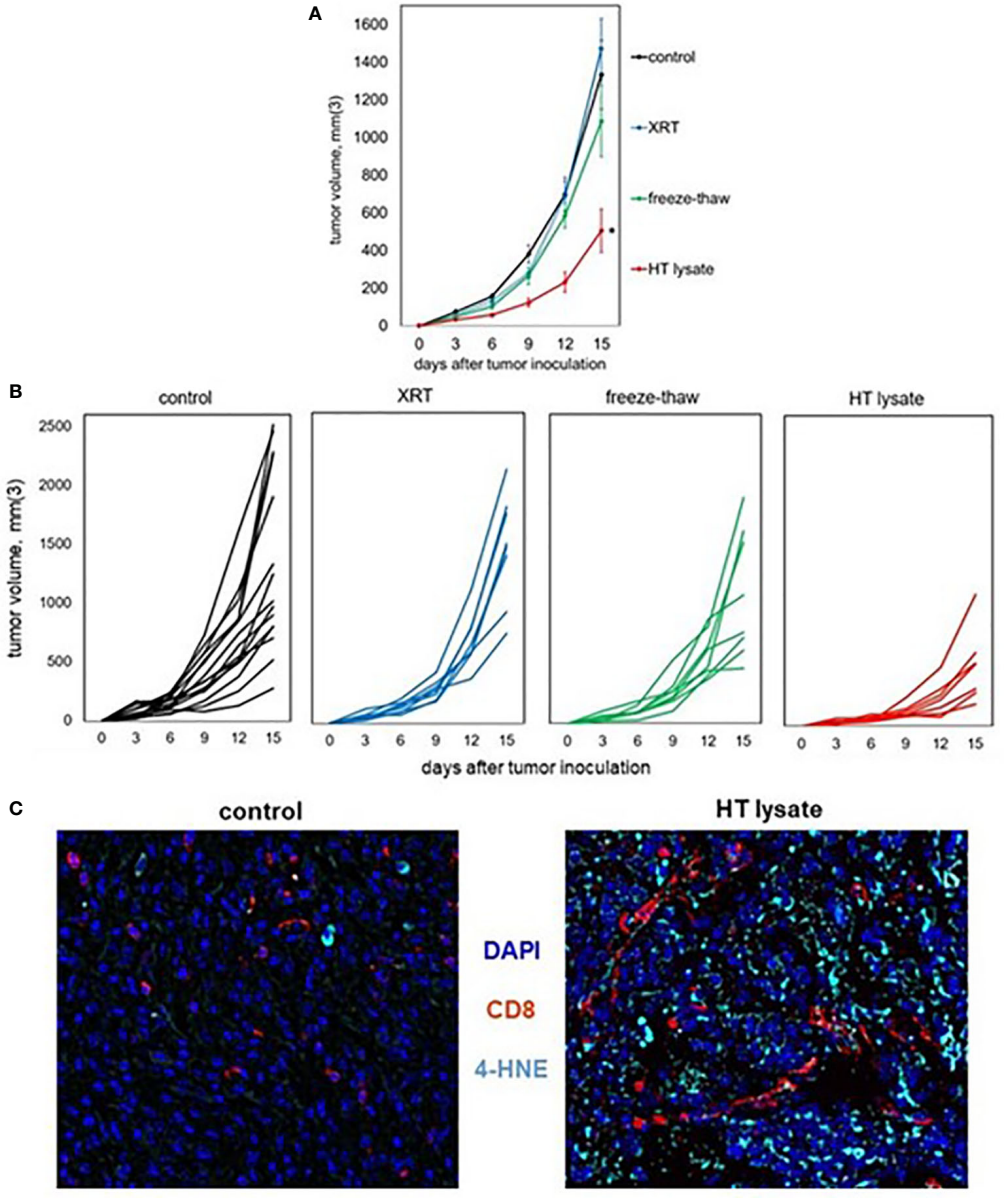
Tumor lysates generated by histotripsy confer partial protection when administered as vaccines. Mice bearing B16F10 tumors were treated with no therapy, 15 Gy radiation therapy (XRT), or histotripsy (“HT”) ablation on day 9. Tumors were explanted on day 10 and untreated tumors were dissociated by 3 cycles of alternative freezing and thawing. The resulting lysates were centrifuged to generate cell-free fractions, and fractions were delivered *via* intraperitoneal injection into naïve mice one day prior to B16F10 flank injection. **(A, B)** Whereas vaccination with tumor lysates generated by XRT or freeze-thaw conferred no protection against B16F10 challenge tumor growth as compared with unvaccinated controls, mice receiving tumor lysates generated by histotripsy exhibited slower challenge tumor growth kinetics. **(C)** Multicolor immunohistochemistry of challenge tumors growing in unvaccinated control and HT lysate-vaccinated mice showed marked increases in both CD8+ cell infiltration and 4-HNE accumulation. (n=3-6 mice per group; *=p<0.05 compared with controls).

To experimentally confirm the ability of histotripsy to promote immunogenic cell death, vaccines were prepared from mice bearing unilateral B16F10 melanoma tumors that were treated with no therapy (control), 15 Gy radiation therapy, or histotripsy tumor ablation 10 days after tumor inoculation. Vaccines were generated from tumors excised one day after treatment. Untreated control tumors were exposed to three cycles of alternating freezing and thawing. Tumors were mechanically dissociated and centrifuged, and cell-free supernatants were administered *via* intraperitoneal injection into naïve mice one day prior to flank inoculation with B16F10 challenge tumors. As shown in **Figures 7A, B**, only mice receiving vaccines generated by histotripsy ablation demonstrated measurable inhibition of challenge tumor growth. Moreover, multicolor immunohistochemistry revealed significant 4-HNE accumulation among B16F10 tumors in mice that received histotripsy-generated vaccines but not in control unvaccinated mice (**Figure 7C**). These results support the observation that histotripsy tumor ablation induces immunogenic cancer cell death.

## Discussion

Growing tumors exert immunosuppressive influences that thwart anti-tumor immune responses (48–54). There has been longstanding interest in the capacity of ablation to activate tumor-directed immune responses. Measurable and occasionally abscopal anti-tumor immune responses have been observed with cryoablation, radiofrequency ablation and radiation, largely when used in combination with immunomodulatory agents like CpG or pro-inflammatory cytokines (1–13). We recently reported that histotripsy, a non-thermal mode of mechanical FUS ablation (32–36), stimulates potent local and abscopal CD8+ T cell infiltration and tumor inhibition as monotherapy; using murine models of melanoma and hepatocellular carcinoma, we observed that this effect can enhance checkpoint inhibition immunotherapy (37). In this report, we applied microscopic and other assays to these same murine models to record the spatiotemporal evolution of local and abscopal immune response in treated tumors and in distant, untreated tumors.

We observed that the abscopal effect of histotripsy was more pronounced in the Hepa1-6 hepatocellular carcinoma model than in the B16 melanoma model. Unlike exponentially-growing B16F10 tumors, Hepa1-6 tumors exhibit a more linear tumor growth pattern, and infiltrating immune cell populations and responsiveness to CI immunotherapy suggest that Hepa1-6 tumors may be more immunogenic than B16F10 tumors (55, 56). These findings suggest that the abscopal effects induced by histotripsy monotherapy are not limited to melanoma tumors, and that the magnitude of these effects may be greater when applied to intrinsically immunogenic tumors. In addition, the absence of abscopal tumor inhibition in mice with antigenically discordant tumors (i.e., normal growth of non-ablated B16F10 tumors after contralateral ablation of immunogenic Hepa1-6 tumors) underscores the antigenic specificity of this effect.

To overcome the potent immunosuppressive influences exerted by growing cancers, immunostimulatory therapies should ideally trigger immunogenic cell death pathways that liberate tumor antigens within a pro-inflammatory context of immunological “danger”. Unlike non-immunogenic cell death, in which cells undergo involutional apoptotic demise within an anti-inflammatory, tolerogenic cytokine milieu, immunogenic cell death pathways are programmed responses to triggers such as severe endoplasmic reticulum stress that share a common feature of DAMP translocation (46, 47, 57–67). The pro-inflammatory properties of DAMPs engender the infiltration and activation of APCs to process and present tumor antigens for the eventual priming of adaptive immune responses – effectively initiating the cancer immunity cycle (14). We previously reported that histotripsy tumor ablation results in the widespread translocation of DAMPs like HMGB1 and calreticulin within the ablation zone (37). In this report, RNASeq analyses of tumor cell populations at early time points detected severe cellular and ER stress transcriptional responses to partial histotripsy ablation. We also measured profound increases in TNFα within ablated tumors immediately following histotripsy. Given the nature of tissue cavitation induced by histotripsy, some of the early DAMP release seen in ablation zones could be from mechanical disruption of cellular and nuclear membranes. However, morphologically-intact tumor cells along the periphery of the mechanical ablation zone appear to undergo necroptosis, a catastrophic TNFα-driven immunogenic programmed cell death pathway culminating in the formation of cell membrane pores that disrupt cellular integrity and release DAMPs. RNASeq confirmed significant upregulation of transcriptional pathways associated with necroptosis. The immunostimulatory consequences of necroptosis are well-documented, and active investigations are underway to harness the ability of specific chemotherapeutic agents like doxorubicin to induce necroptosis of cancer cells (46, 57).

Concordant with the ability of DAMPs to engage APC pattern recognition receptors to trigger antigen processing and presentation, the massive release of DAMPs within the ablation zone is followed by a rapid influx of myeloid APC populations. Indeed, infiltrating immune cell populations at early time points following histotripsy are characterized by transcriptional indicators of innate immune response activation. This response is transient, as these myeloid cell populations are largely absent from ablated tumors 3 days after histotripsy. However, we observe evidence of enhanced APC:T cell interaction within tumor-draining lymph nodes after histotripsy. These events are followed by the trafficking of T cells into non-ablated zones of treated tumors and distant non-ablated tumors, coinciding with abscopal tumor growth inhibition. Notably, CD8+ T cell infiltration is preceded and accompanied by the infiltration of NK cells, with early mobilization of NK cells in both treated and contralateral abscopal tumors. This early abscopal NK cell response may explain our observation of almost immediate abscopal inhibition of tumor growth following unilateral histotripsy tumor ablation.

At later time points, we observed gradual increases in HMGB1 translocation within non-ablated zones of treated and abscopal tumors, with a close spatial correlation between HMGB1 release and CD8+ T cell infiltration; in control tumors, no correlation was observed between the sparse numbers of intratumoral CD8+ T cells and tumoral HMGB1 release. This observation is consistent with the recent demonstration by Minute and co-authors, who observed that tumor cells exposed to activated tumor antigen-specific CD8+ T cells undergo immunogenic cell death as evidenced by HMGB1 and calreticulin translocation (68). Ferroptosis is a pathway of immunogenic cell death characterized by iron-dependent lipid peroxidation and HMGB1 release that may have particular therapeutic importance in oncology, as cancer cells of numerous histological types are known to be uniquely susceptible to ferroptosis (15–20, 67). In both histotripsy-treated and histotripsy-abscopal tumors, we observed a very strong spatial correlation between CD8+ T cell infiltration and tumor cell accumulation of 4-HNE, which is a metabolic byproduct of ferroptosis. In control tumors, cancer cells in areas of sparse CD8+ T cell infiltration exhibited no 4-HNE accumulation. Recent work from Yang and co-authors showed that cancer cell ferroptosis-inducing adjuvants enhanced the efficacy of radiofrequency ablation, but that radiofrequency ablation alone did not induce ferroptosis (10). Notably, Wang and co-authors recently demonstrated that ferroptosis is the mechanism of cell death by which checkpoint inhibition-activated CD8+ T cells exert their cytotoxic effect on cancer cells (18). Our data raise the possibility that histotripsy could be priming CD8+ T cells to kill cancer cell targets using the same cytotoxic mechanism induced by checkpoint inhibition. Indeed, we found that the therapeutic cooperativity previously observed between checkpoint inhibition and the abscopal effects of histotripsy is associated with a synergistic accumulation of cancer cell ferroptotic byproducts. The delayed release of HMGB1 and accumulation of 4-HNE (seen with ferroptosis) that occurs in tumor regions distant from the ablation zone are temporally and spatially distinct from the early release of HMGB1 and co-localization of pRIPK3 and pMLKL (seen with necroptosis) that occurs in tumor regions adjacent to the ablation zone. We observed that *in vitro* co-culture with CD8+ T cells harvested from histotripsy-treated melanoma-draining lymph nodes directly led to accumulation of 4-HNE in melanoma cells – an effect not seen following co-culture with CD8+ T cells harvested from untreated melanoma-draining lymph nodes. However, ongoing and future work using CD8+ T cell-deficient models, direct quantitation of lipid peroxidation, and metabolic inhibitors of ferroptosis will be needed to determine if CD8+ T cells and ferroptotic cell death pathways are mechanistically necessary for the abscopal immune effects associated with histotripsy tumor ablation.

The experimental “gold standard” demonstration of immunogenic cell death is the ability of treated tumor debris to function as immunoprotective vaccines (69, 70); our observation that mice immunized with tumor homogenates generated by histotripsy (but not radiation or thermal dissociation) are partially resistant to challenge tumor growth supports the likelihood that histotripsy tumor ablation induces immunogenic cell death. This immunoprotection was not complete, as vaccination with histotripsy-generated tumor homogenates did not confer immunity to challenge tumor growth. However, more potent vaccination strategies (e.g., using pulsed dendritic cells) could enable the development of histotripsy-derived personalized anti-tumor vaccines. Of note, we challenged mice with tumor only one day after tumor homogenate injection; it is possible that a longer interval of time might have enabled more substantive immune protection for tumor homogenates generated by histotripsy, as well as by freeze/thaw and radiation. Of note, we previously observed that tumor homogenates generated by histotripsy ablation but not freeze/thaw dissociation contain immunogenically intact tumor antigens capable of stimulating tumor antigen-specific CD8+ T cells (37); thus, inoculation of intact tumor antigens could have contributed to the ability of histotripsy tumor homogenates to promote partial immune protection even in the absence of a more prolonged post-vaccination latency period. Our observation of CD8+ T cell infiltration and 4-HNE accumulation in challenge tumors growing in mice pre-treated with histotripsy-generated tumor vaccines suggests that early immunogenic cell death induced by histotripsy could be sufficient for triggering potent systemic anti-tumor adaptive immune responses. Ongoing studies are investigating the degree to which the subcellular and cellular events that follow histotripsy ablation, including the early induction of necroptosis and the late induction of CD8+ T cell-associated ferroptosis, could be mechanistically responsible for the abscopal effects of histotripsy focused ultrasound ablation.

## Data availability statement

The datasets presented in this study are deposited in the GEO repository, accession number GSE221448.

## Ethics statement

The animal study was reviewed and approved by Institutional Animal Care and Use Committees of the VA Ann Arbor Healthcare System and University of Michigan.

## Author contributions

Mouse work and experiments were performed by AP, RM, JG, AF, HG, JD, HC, NB, and AG. Protein sequencing was performed by RM and AG, and RNA sequencing was performed by RM and JG. Histotripsy tumor ablations were performed by RM, RH, and TW, and microscopy assays were performed by AP, RM, and AG. *In vitro* co-culture experiments were performed by BS, RM, and AG. Data analysis was performed by MO, ZX, AG, and CC, and manuscript writing was performed by AP, RM, JG, and CC. All authors contributed to the article and approved the submitted version.
