# Supplementary figures and images for: Spatiotemporal local and abscopal cell death and immune responses to histotripsy focused ultrasound tumor ablation
Source: Front Immunol. 2023 Jan 23;14:1012799. doi: 10.3389/fimmu.2023.1012799 (PMC9900174; doi:10.3389/fimmu.2023.1012799)

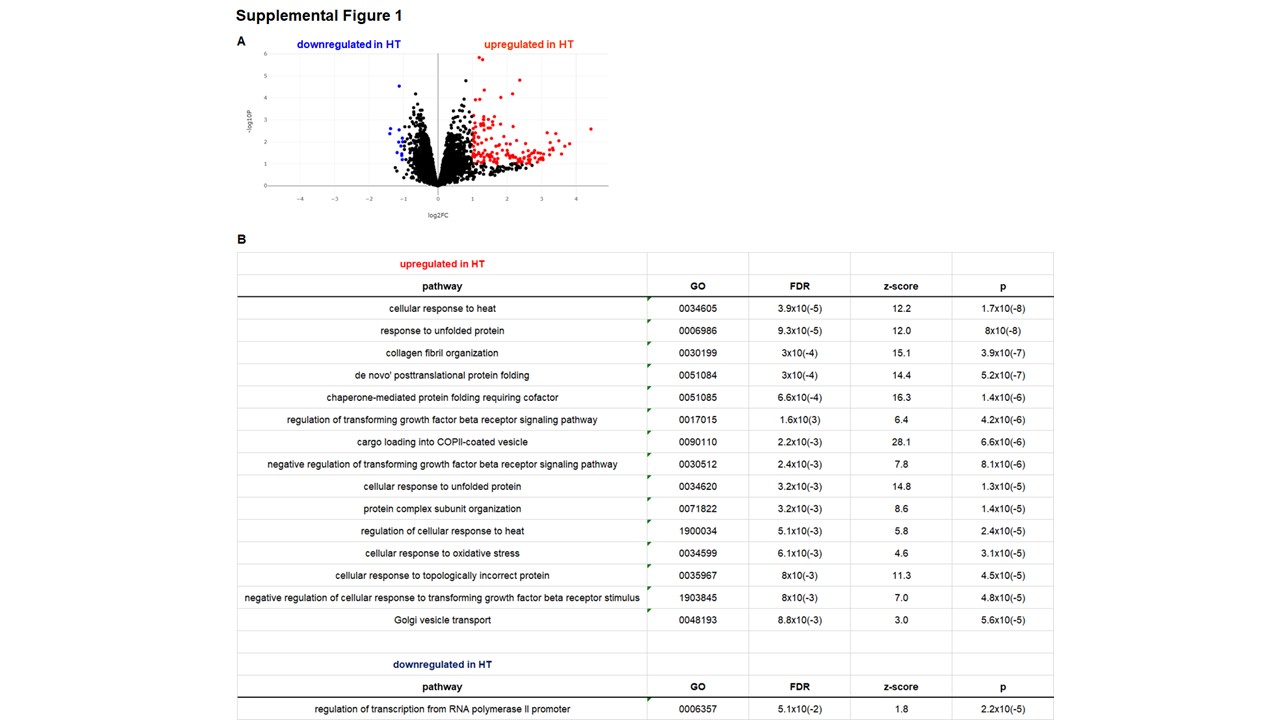

Supplement: Supplementary file 1 [file Image_1.jpeg]

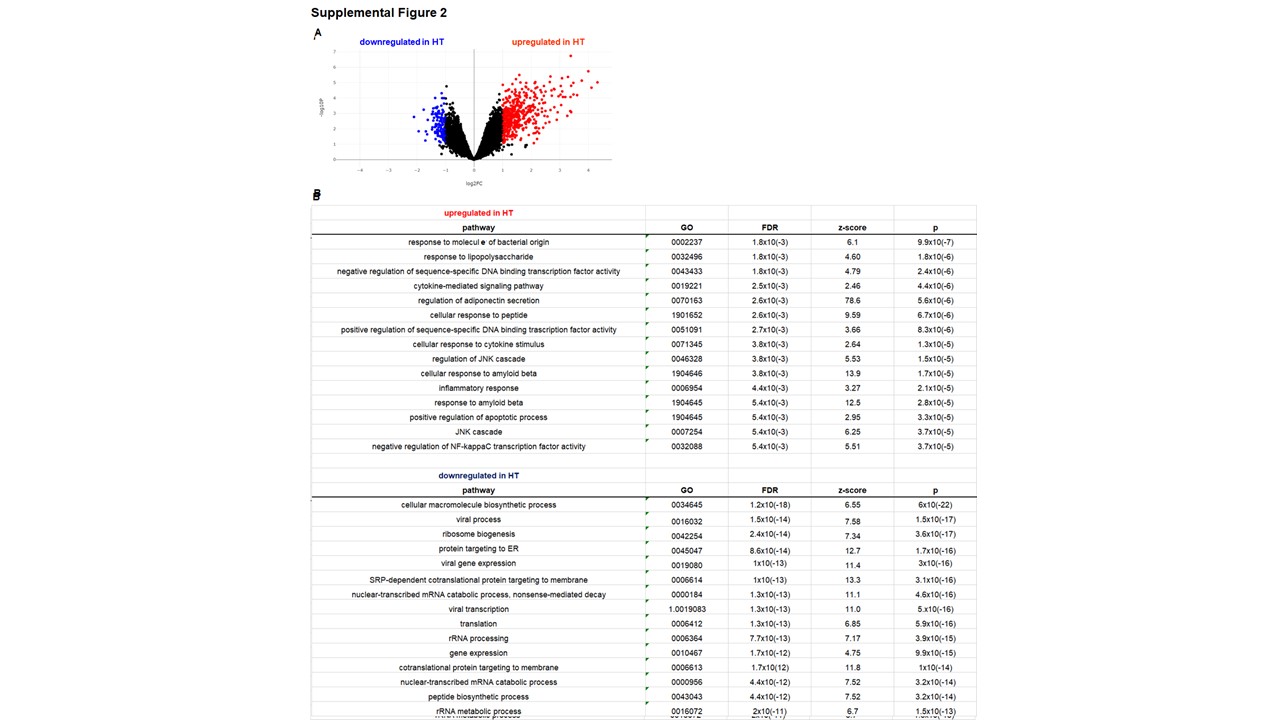

Supplement: Supplementary file 2 [file Image_2.jpeg]

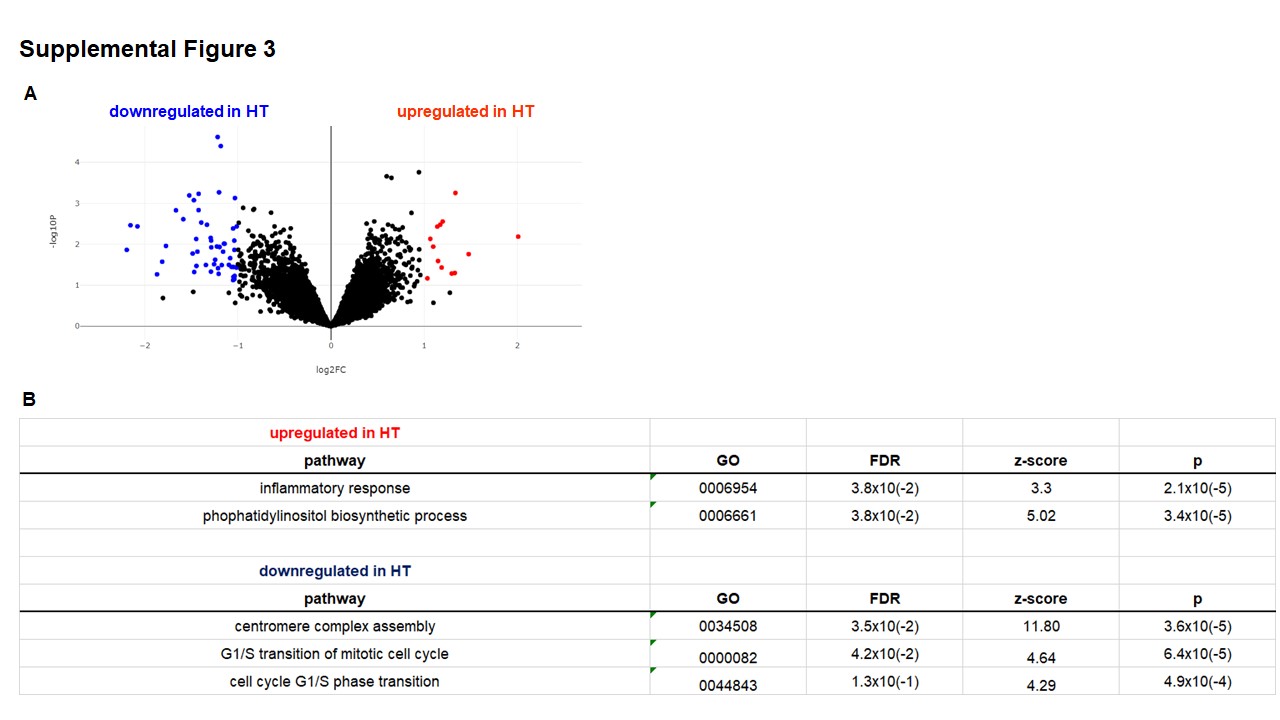

Supplement: Supplementary file 3 [file Image_3.jpeg]

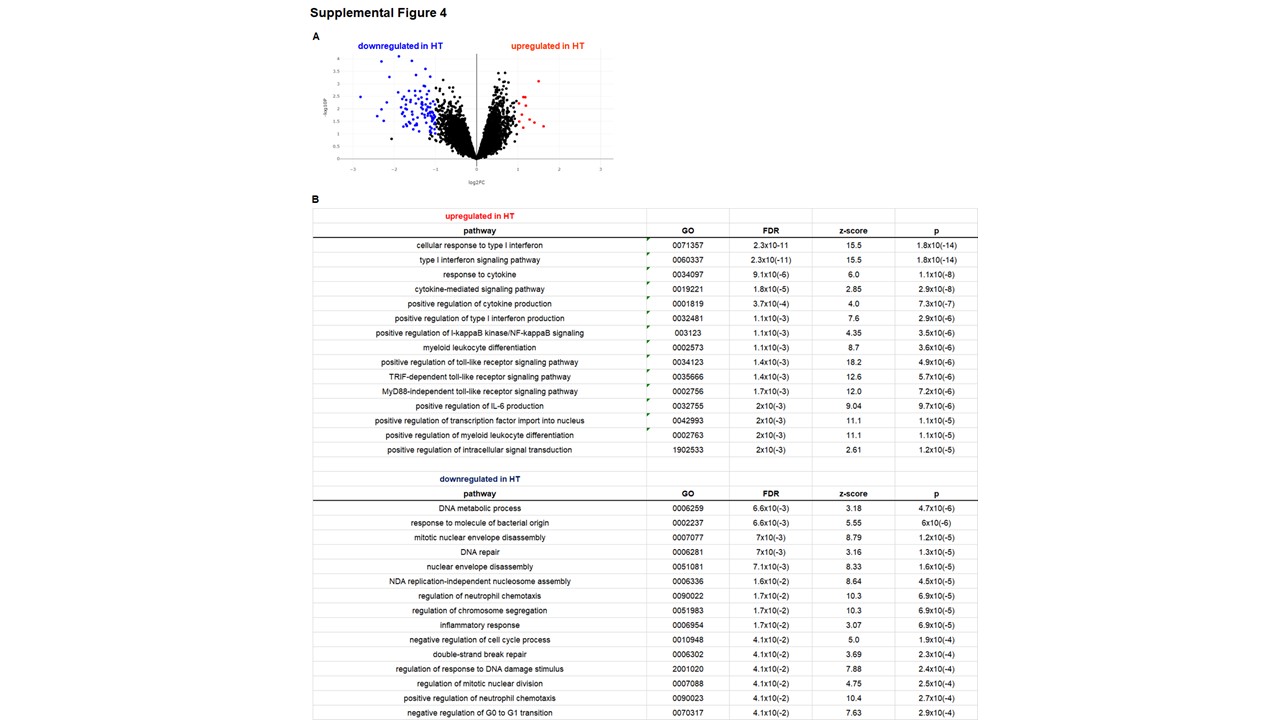

Supplement: Supplementary file 4 [file Image_4.jpeg]

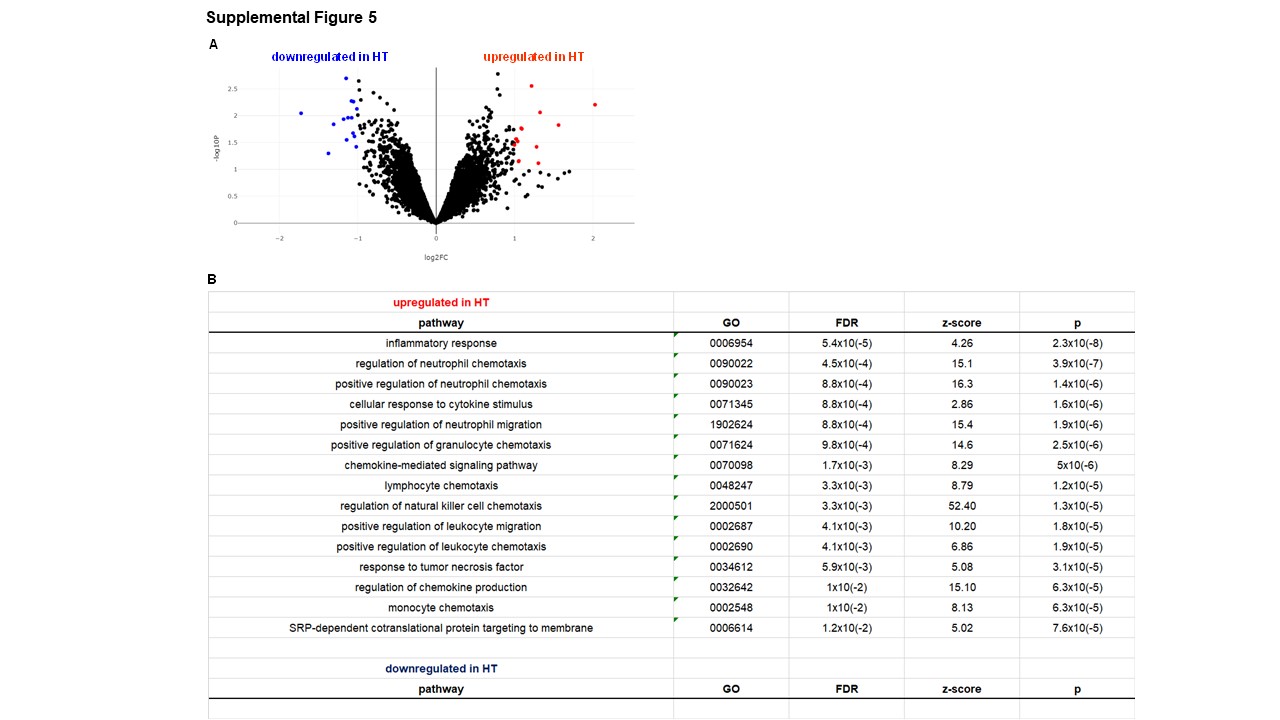

Supplement: Supplementary file 5 [file Image_5.jpg]
